# Epidermal Permeability Barrier in the Treatment of Keratosis Pilaris

**DOI:** 10.1155/2015/205012

**Published:** 2015-02-24

**Authors:** Tanawatt Kootiratrakarn, Kowit Kampirapap, Chakkrapong Chunhasewee

**Affiliations:** Institute of Dermatology, Bangkok 10400, Thailand

## Abstract

*Objectives*. To evaluate and compare the efficacy, safety, hydrating properties, and tolerability of 10% lactic acid (LA) and 5% salicylic acid (SA) in the therapy of keratosis pilaris (KP). *Material and Method*. Patients with KP were randomized for treatment with either 10% LA or 5% SA creams being applied twice daily for 3 months. The patients were clinically assessed at baseline and after 4, 8, and 12 weeks of treatment and 4 weeks after treatment. The functional properties of the stratum corneum (SC) were determined before treatment, 12 weeks, and follow-up phase by high-frequency conductance and transepidermal water loss (TEWL). *Results*. At the end of the trial, the mean reduction of the lesions from baseline was statistically significant for 10% LA (66%) and 5% SA (52%). During the treatment, higher conductance values were found on both group and this improvement was maintained until the follow up period. No significant differences in transepidermal water loss were observed after treatment. The adverse effects were limited to mild irritation localized on the skin without systemic side effect. *Conclusion*. The study demonstrated that 10% LA and 5% SA are beneficial to treat KP with the significantly clearance and marked improvement as by instrumental evaluation.

## 1. Introduction

Keratosis pilaris (KP) is a common benign disorder that presents an eruption of symmetrically distributed, keratotic follicular papules on the proximal extremities of young individuals. Although it is neither life-threatening nor physically debilitating, it can severely affect those individuals socially and psychologically. Furthermore, commonly employed treatments appear to show a lack of satisfactory efficacy. These medications have included an emollient cream and keratolytic agents containing lactic acid or salicylic acid [1]. In the present study, we evaluated the clinical outcome as well as the functions of the stratum corneum (SC) by employing a prospective split side-controlled trial of lactic acid and salicylic acid before and after topical treatment with representative keratolytic agents.

## 2. Material and Method

### 2.1. Patients and Procedures

A prospective, randomized, and clinical study comparing 10% lactic acid and 5% salicylic cream was carried out at the Institute of Dermatology, Bangkok, Thailand. The protocol was approved by the Ethical Committee of the Institute of Dermatology and written informed consent was obtained from all participants.

The patients had a clinical diagnosis of keratosis pilaris, which showed the extensive keratotic follicular papules, almost entirely on the extensor and lateral aspect of proximal extremities, symmetrically. Fifty participants were enrolled in this clinical study. Patients were in good health and free of other skin disease or physical condition that would impair evaluation of treatment areas. Subjects were ineligible to participate if they (i) were sensitive to the ingredients in the formulations; (ii) were pregnant or lactating; (iii) had used topical therapy (emollients, moisturizers, corticosteroids) within 1 month prior to the study; (iv) were receiving concurrent systemic therapy with corticosteroids or retinoid. They were allowed to continue their usual daily activities throughout the trial.

### 2.2. Randomized Treatment Phase

The patients were enrolled in this study to evaluate the effect of 10% lactic acid versus 5% salicylic acid cream. The vehicle has been controlled by using base cream (8% stearyl alcohol, 4% cetyl alcohol, 18% liquid paraffin, 5.7% propylene glycol, 2% white beeswax, 5.5% sodium lauryl sulfate, and 1% paraben conc.) with active gradient 10% lactic acid or 5% salicylic acid, respectively. They were told to apply the one designated agents twice daily on each of their extensor upper arms by using one hand to apply one test medication to the opposite upper arm and vice versa. The information stored securely for each patient and examining dermatologists were blinded to this information. Patients were reexamined by the dermatologist at 4, 8, and 12 weeks after beginning the study and 4 weeks follow-up phase and any changes in number of lesions were documented in questionnaires designed for this purpose.

### 2.3. Subjective Self-Assessment

Self-assessments were solicited by questionnaire; study participants were interviewed and questioned regarding therapy benefits. An open-ended question asked patients if any treatment had been successful and, if so, to evaluate overall change in smoothness and dark spot of the affected area. The quantification of any amelioration in their KP was also done by marking the improved percentage from baseline.

### 2.4. Investigator Assessment

Investigators' evaluations were recorded via questionnaires by three independent dermatologists. At the end of treatment and follow-up phase, the change in the signs of papules and postinflammatory pigment alteration were evaluated in the overall disease severity by percent of improvement.

### 2.5. Biophysical Skin Parameters Obtained by Noninvasive Measurements

High-frequency conductance, which is a parameter for the hydration state of the skin surface, was measured with a skin surface hygrometer (Skicon 200, IBS Ltd., Hamamatsu, Japan) with a probe of 2 mm inner diameter and 4 mm outer diameter electrodes. Transepidermal water loss (TEWL), a parameter for the water barrier function of the stratum corneum SC, was measured with close-chamber evaporimeter (Vapometer, Delfin Technologies Ltd., Kuopio, Finland) [2]. All measurements were performed on the mid portion of the upper arm by a 30 min acclimatization time in a climate chamber, in which the room temperature and relative humidity were adjusted to 25°C and the relative humidity was 65 to 70%, respectively. The conductance and TEWL were recorded at the baseline, week 12 of treatment and 4-week follow-up after complete treatment.

### 2.6. Statistical Analysis

Statistical analyses were performed with SPSS 16 for Windows software (SPSS Inc., Chicago, IL, USA). Scores of the clinical assessment were compared between each treated side using the Wilcoxon signed-ranks test. The values of the conductance, TEWL, were compared between each treatment with the paired *t*-test. *P* values of <0.05 were accepted as statistically significant.

## 3. Result

The age of onset of KP was as follows; during first decade in 57%, second decade 31%, and third decade 12%. Body sites affected included the arms (93%), legs (26%), face (6%), and buttocks (27%). Family history of KP was found in 67%. An associated atopic condition (allergic rhinitis, asthma, and eczema) was recorded in 42%. No seasonal variation was noted in our patients. Half of them felt that their skin was dry and considered that dry environment was a precipitating factor. Most of patients (65%) had previously sought and received treatments and felt dissatisfaction with the appearance, embarrassment, and lack of self-confidence. Social dysfunction has also been observed, including concerns about social appearances in public.

**Figure 1:**
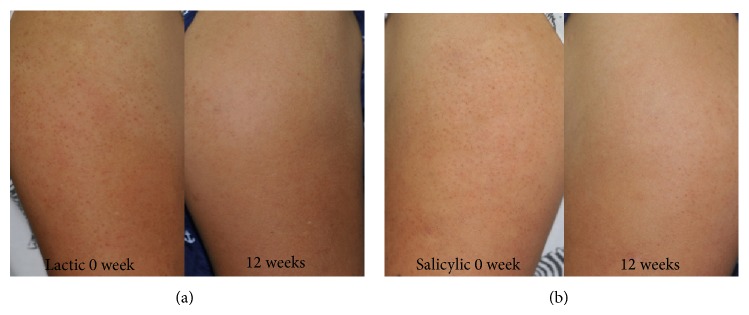
Clinical appearance of keratosis pilaris before and after 12 weeks of treatment with 10% lactic cream (a) and 5% salicylic cream (b). They show decreased hyperkeratotic papules at the end of trial.

**Figure 2:**
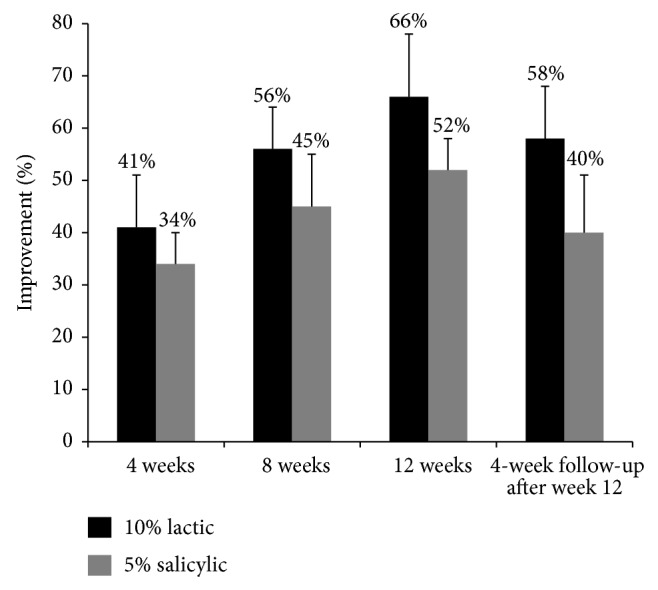
Compared with the baseline, both treatments showed a statistically significant reduction of the lesions at the end of 4, 8, and 12 weeks of treatment (*P* < 0.05). The observed rate of decline was greater during the first 4 weeks, becoming more gradual during the next 4 weeks until the end of 12 weeks. The mean percentage of reduction from the baseline up until the end of the trial was 66% for the lactic acid group in contrast to 52% for the salicylic acid group. These changes from the baseline until the end of the 12 weeks were all statistically significant in both groups (*P* < 0.05).

Fifty participants completed the monthly assessment for evaluation of treatment efficacy (Figure 1). Compared with baseline, both treatments showed a statistically significant reduction of lesions at the end of 4, 8, and 12 weeks of treatment (*P* < 0.05). The rate of decline was greater during the first 4 weeks and more gradually decreased during the next 4 weeks until the end of 12 weeks. The mean percentage of reduction from baseline up until the end of the trial was 66% in the lactic acid group and 52% with the salicylic acid group. The change from baseline until the end of the 12 weeks was statistically significant in both groups (*P* < 0.05). The mean difference between both groups from baseline until the end of 12 weeks was 14% which was statistically significant (*P* < 0.05), the lactic acid group was more effective than the salicylic acid group (Figure 2).

The mean percentage change forming baseline to up until the 4 and 8 weeks was 41% and 56% in the lactic acid group and 34% and 45% in the salicylic acid group, respectively. Both treatment groups had a statistically significant reduction in the mean percentage improvement from baseline until the end of the 4 and 8 weeks (*P* < 0.05).

The patients in both groups assessed the aesthetic features of both creams. The greasiness of both creams was similar; however, the lactic acid group complained more about malodor and irritation when applying the cream. The adverse events reported during the present study showed only irritation, which was typically slightly burning or itching sensation with no visible reaction on the skin. These reactions were found more common in the lactic acid group than the salicylic acid group but the difference was not statistically significant.

The instrumental assessments were summarized in Table 1. Although at the end of 12-week period showed no statistically significant changes in TEWL in either lactic acid or salicylic acid treated sites as compared with their data obtained before treatment, the skin conductance became significantly higher after treatment with both agents, as compared with their baseline values. These obtained data also showed the sustainable benefits even at 4-week follow-up phase.

## 4. Discussion

Keratosis pilaris is known to be a common disorder which affects 40% of population [3]. However, the disease has been poorly documented in the literature. The isolated form frequently appears in childhood with remission by adulthood in many patients. Most of our patients developed KP during the first decade of life, which agrees with the previous finding that KP commences in childhood and reaches its peak prevalence during adolescence. A family history of KP in 67% would support and agree with an autosomal dominant mode of inheritance. The families with incomplete penetrance had been postulated [4]. The increasing prevalence of KP in autosomal dominant ichthyosis had also been reported.

Patients' tolerance to the rough texture and cosmetic appearance of KP varies considerably and is frequently discordant with the clinical signs [5]. The interaction of KP and psychosocial issues is complex and, in adolescents, can be associated with developmental issues of body image, socialization, and sexuality [6, 7]. However, the study on the psychosocial impact of KP has not been documented. The impact of KP may have been underestimated. Our data showed that 40% of those with KP have significant effect on self-image and impacts in the quality of life; therefore, the effective treatment should result in the improvement of anxiety, depression, and body satisfaction.

The treatment usually begins with reassurance for the patient and a discussion of general skin care. Measures should be taken to prevent excessive skin dryness, such as decreasing the frequency of skin cleansing, brief water showers, and using mild soaps. The keratolytic agent such as one that contains lactic acid, salicylic acid, or urea may be beneficial. Lactic acid functions, primarily, as modulator of skin keratinization, although it also is referred to as humectant, pH adjuster, and mild irritation [8]. The application should show the reduction of corneocyte adhesion at the lowest levels of the stratum corneum, which results in desquamation of both normal and diseased skin, also resulting in normalization of retention hyperkeratosis. With continued treatment, the lactic acid 10% treatment site demonstrated a dramatic increase in turnover rate [9, 10]. Salicylic acid is a topical keratolytic agent which is believed at act by reducing cohesion between keratinocytes. Symptoms of toxicity form percutaneous absorption and contact sensitizer have been reported [11]. The urea cream usually produces keratolytic effect without the stimulatory response to basal cells, avoiding the production of excess or abnormal corneocytes [12]. We particularly like a cream preparation consisting of 10% lactic acid or salicylic acid. We hypothesize that, in the differences, mechanism of action may explain the differences seen in the two treatments. A follow-up study also may be desirable to study this important variable.

The present study confirmed the effectiveness of 10% lactic acid and 5% salicylic acid in the treatment of KP. Both medications showed greater statistically significant improvement of KP at the end of 4 weeks. However, the data indicated that clinical observation ratings by patients differed between the two treatments. Most measures showed that improvement was achieved in less time with 10% lactic acid cream than with 5% salicylic acid cream. Moreover, patients receiving 10% lactic acid showed rapid onset of improvement from baseline at the end of 4 weeks and sustained improvement afterwards until the end of 12 weeks. Furthermore, the investigators' overall assessment showed over 62.5% of patients treated with 10% lactic acid achieved marked improvement and clearance at the end of 12 weeks, which showed the high efficacy of this treatment. However, patients treated by 10% lactic acid showed mild local irritation as a side effect that was well tolerated.

The cutaneous bioengineering was also used to evaluate the mechanical and functional characteristics of the skin affected by KP. The most commonly used methods for evaluating moisturization are cutaneous electrical capacitance and TEWL. Cutaneous electrical capacitance involves the passage of low-frequency alternate current through the skin, whose electrical conductivity depends on the water contents of the SC and its integrity. TEWL determines the flow of evaporation of water through the SC, thus allowing evaluation of its barrier function. We found an increase in the hydration state of the SC when evaluated with the measurements of high-frequency conductance of the treated skin at the end of treatment. Conductance findings suggested that 10% lactic acid cream improves texture by the stimulation of cell growth upward towards the skin surface, which might produce outer skin hydration at a slower pace. Thus, it can be estimated that at end of treatment it showed greater hydration. Five percent salicylic acid cream showed improvement of hydration by directly removing the upper surface layer of dead cells, thereby softening the skin. This suggests that both may significantly decrease SC cohesion by only minimally disrupting skin barrier to water diffusion. Since corneodesmosomes are believed to be the major component providing SC cohesion [12, 13], our results indicate that both treatments mainly affect their structure and seems not significantly to perturb the SC lipid composition and organization, which is mainly responsible for SC excellent barrier properties.

In conclusion, the clinical observation ratings by patients and investigators, as well as instrument measures, showed the effectiveness of the two treatments. The conclusion from the present study is that 10% lactic acid should be chosen as standard treatment for KP in preference to salicylic acid in the view of its higher efficacy. When this therapy was used for distressing or extensive keratosis pilaris, it has been observed to be effective, convenient, well-accepted, well-tolerated regimen.

## Figures and Tables

**Table 1:** At the end of the 12-week treatment period, the obtained skin conductance values were significantly higher for each of the therapies, as compared with their respective baseline values. These obtained values were higher for the 10% lactic acid cream treated side than the 5% salicylic acid treated side. By contrast, TEWL showed no statistically significant changes in both lactic acid and salicylic acid-treated sites as compared with those values measured before treatment.

	Conductance (±SD)		TEWL (±SD)	
	10% lactic	5% salicylic	10% lactic	5% salicylic
Base line	121.5 ± 60.0	124.5 ± 55.3	8.1 ± 1.6	7.9 ± 1.8
Week 12	169.1 ± 74.0^*^	148.5 ± 49.9^*^	7.7 ± 1.6	7.7 ± 1.4
4-week follow-up	155.3 ± 53.2^*^	140.6 ± 43.6^*^	7.5 ± 2.3	7.7 ± 1.2

^*^ *P* < 0.05.
